# Analysis of clinical characteristics and laboratory findings of 95 cases of 2019 novel coronavirus pneumonia in Wuhan, China: a retrospective analysis

**DOI:** 10.1186/s12931-020-01338-8

**Published:** 2020-03-26

**Authors:** Gemin Zhang, Jie Zhang, Bowen Wang, Xionglin Zhu, Qiang Wang, Shiming Qiu

**Affiliations:** 1 Department of infectious disease, Wuhan Xinzhou District People’s Hospital, Wuhan, China; 2 Department of Neurology, Wuhan Xinzhou District People’s Hospital, 61 Xinzhou street, Xinzhou District, Wuhan, 430000 Hubei Province China; 3 Department of critical care medicine, Wuhan Xinzhou District People’s Hospital, Wuhan, China

**Keywords:** 2019 novel coronavirus, Pneumonia, Clinical characteristics, Laboratory findings

## Abstract

**Background:**

Since December 2019, 2019 novel coronavirus pneumonia emerged in Wuhan city and rapidly spread throughout China and even the world. We sought to analyse the clinical characteristics and laboratory findings of some cases with 2019 novel coronavirus pneumonia .

**Methods:**

In this retrospective study, we extracted the data on 95 patients with laboratory-confirmed 2019 novel coronavirus pneumonia in Wuhan Xinzhou District People’s Hospital from January 16th to February 25th, 2020. Cases were confirmed by real-time RT-PCR and abnormal radiologic findings. Outcomes were followed up until March 2th, 2020.

**Results:**

Higher temperature, blood leukocyte count, neutrophil count, neutrophil percentage, C-reactive protein level, D-dimer level, alanine aminotransferase activity, aspartate aminotransferase activity, α - hydroxybutyrate dehydrogenase activity, lactate dehydrogenase activity and creatine kinase activity were related to severe 2019 novel coronavirus pneumonia and composite endpoint, and so were lower lymphocyte count, lymphocyte percentage and total protein level. Age below 40 or above 60 years old, male, higher Creatinine level, and lower platelet count also seemed related to severe 2019 novel coronavirus pneumonia and composite endpoint, however the *P* values were greater than 0.05, which mean under the same condition studies of larger samples are needed in the future.

**Conclusion:**

Multiple factors were related to severe 2019 novel coronavirus pneumonia and composite endpoint, and more related studies are needed in the future.

## Background

The 2019 novel coronavirus pneumonia is caused by a novel betacoronavirus that is currently named 2019 novel coronavirus, which was identified by deep sequencing analysis from lower respiratory tract samples [1, 2]. The 2019 novel coronavirus is the seventh member of enveloped RNA coronavirus that can infect humans [3]. On one hand most human coronavirus infections are mild, on the other hand the infections of coronavirus including 2019 novel coronavirus, severe acute respiratory syndrome coronavirus (SARS-CoV) and Middle East respiratory syndrome coronavirus (MERS-COV) could be severe or even deadly [3].

In early December 2019, the first patient with 2019 novel coronavirus pneumonia emrged in Wuhan city, Hubei province, China [1]. Subsequently the 2019 novel coronavirus pneumonia rapidly spread throughout china. Soon afterwards many countries declared the first case of 2019 novel coronavirus pneumonia [4–8]. The 2019 novel coronavirus pneumonia emerged in more than 151 countries, and the number of laboratory-confirmed cases reached 167,511 and that of the death cases was 6606 in the world as of March 16th, 2020 according to the information from the official website of World Health Organization.

In the past 2 months several studies have described the clinical characteristics of patients with 2019 novel coronavirus pneumonia [2, 9, 10]. However some cases in these studies had one or more chronic or severe underlying diseases, which made it difficult to fully assess the role of 2019 novel coronavirus in 2019 novel coronavirus pneumonia without too much interference. Thus we excluded cases with chronic or severe underlying diseases (i.e., chronic lung disease, chronic heart disease, chronic liver disease, chronic kidney disease) from our study and analysed the data on 95 patients with 2019 novel coronavirus pneumonia.

## Methods

### Data sources

We performed a retrospective study on the clinical characteristics and laboratory findings of laboratory-confirmed cases with 2019 novel coronavirus pneumonia.

Inclusion criteria:

1. All cases were diagonosed with pneumonia based on the clinical manifestations and abnormal findings of chest X-ray or computed tomography.
2. A confirmed case with 2019 novel coronavirus pneumonia was defined as a positive result to high-throughput sequencing or real-time reverse-transcriptase polymerase-chain-reaction assay for nasal and pharyngeal swab specimens [1].

Exclusion criteria:

1. patients with Common bacteria or viruses associated with community-acquired pneumonia.
2. Patients with chronic or severe underlying diseases.
3. Procalcitonin level > 0.5 ng/ml.

**Fig. 1:**
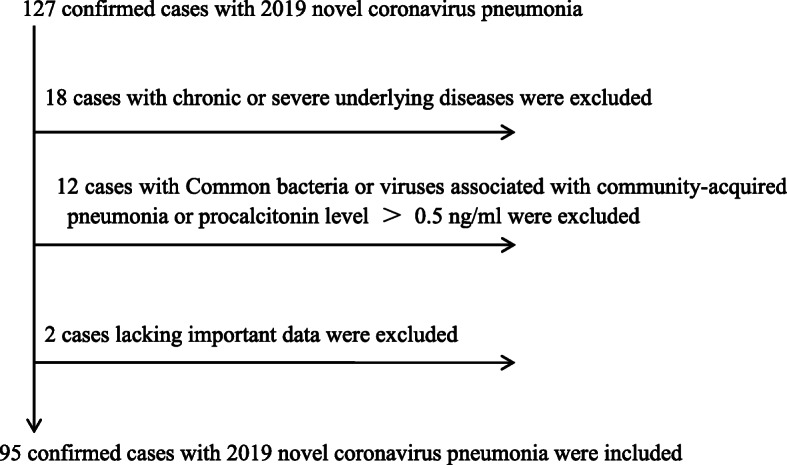
A flow chart, from the total number of patients up to the 95 patients of the study. About 14.2% (18/127) of the patients had one or more chronic or severe underlying diseases, and about 9.4% (12/127) of the patients were co-infected with 2019 novel coronavirus and other respiratory pathogens (i.e., Chlamydia pneumoniae, Mycoplasma pneumoniae, adenovirus and respiratory syncytial virus)

A flow chart, from the total number of patients up to the 95 patients of the study, was shown by Fig. 1. About 14.2% (18/127) of the patients had one or more chronic or severe underlying diseases, and about 9.4% (12/127) of the patients were co-infected with 2019 novel coronavirus and other respiratory pathogens (i.e., Chlamydia pneumoniae, Mycoplasma pneumoniae, adenovirus, and respiratory syncytial virus).

All data including age, sex, temperature and laboratory findings were extracted from electronic medical records. Laboratory assessments consisted of complete blood count, blood chemistry, coagulation test, liver and renal function, serum total protein, C-reactive protein, α-hydroxybutyrate dehydrogenase, lactate dehydrogenase and creatine kinase. The severity of 2019 novel coronavirus pneumonia was defined accroding to the fifth edition diagnosis and treatment program of 2019 novel coronavirus pneumonia issued by the National Health Commission of the People’s Republic of China. Finally, 63 non-severe cases and 32 severe cases were included into our study.

### Definition of severe 2019 novel coronavirus pneumonia

Respiration rate ≥ 30 times / min; at rest, oxygen saturation ≤ 93%; arterial partial pressure of oxygen(PaO2) / fraction of inspired oxygen(FiO2) ≤ 300 mmHg.

### Definition of composite endpoint

The composite endpoint was the admission to intensive care unit (ICU), or mechanical ventilation, or death.

### Laboratory test

Patients usually received lab test every 2 days or when changing in health condition.

### Statistical analysis

Continuous variables were expressed as the medians and interquartile ranges (IQR). Categorical variables were summarized as the counts and percentages in each category. We grouped patients into severe and non-severe cases according to the fifth edition diagnosis and treatment program of 2019 novel coronavirus pneumonia issued by the National Health Commission of the People’s Republic of China. Wilcoxon rank-sum tests were applied to continuous variables, chi-square tests and Fisher’s exact tests were used for categorical variables as appropriate, Kendall rank correlation coefficient (represented by R) was adopted to measure the degree of rank correlation between the fitness and each variable. All analyses were conducted with SPSS software version 23.0 (Statistical Product and Service Solutions). Differences with *P* values < 0.05 were considered significant.

## Results

### Demographic and clinical characteristics

The demographic and clinical characteristics are shown in Table 1.

**Table 1:** Clinical characteristics of 95 patients with 2019 novel coronavirus pneumonia

Clinical characteristics	All patients (***n*** = 95)	Disease severity					Composite endpoint				
		No-severe (***n*** = 63)	Severe (***n*** = 32)	P1	R1	P3	No (***n*** = 70)	Yes (***n*** = 25)	P2	R2	P4
**Age, Median (range)-years**	49.0 (39.0–58.0)	49.0 (41.0–57.0)	50.5 (38.3–58.8)	**0.791**	**0.023**	**0.789**	49.0 (40.8–56.3)	52.0 (37.5–63.0)	**0.413**	**0.070**	**0.410**
**Age groups-No., %**				**0.376**	**−0.026**	**0.794**			**0.120**	**0.022**	**0.820**
<40 years (*n* = 24)	24/95 (25.3)	14/63 (22.2)	10/32 (31.3)				16/70 (22.9)	8/25 (32.0)			
		14/24 (58.3)	10/24 (41.7)				16/24 (66.7)	8/24 (33.3)			
40–60 years (*n* = 54)	54/95 (56.8)	39/63 (61.9)	15/32 (46.9)				44/70 (62.9)	10/25 (40.0)			
		39/54 (72.2)	15/54 (27.8)				44/52 (81.5)	10/52 (18.5)			
>60 years (*n* = 17)	17/95 (17.9)	10/63 (15.9)	7/32 (21.9)				10/70 (14.3)	7/25 (28.0)			
		10/17 (58.8)	7/17 (41.2)				10/17 (58.8)	7/17 (41.2)			
**Age groups-No., %**				**0.192**	**0.143**	**0.164**			**0.061**	**0.203**	**0.049**
40–60 years (n = 54)	54/95 (56.8)	39/63 (61.9)	15/32 (46.9)				44/70 (62.9)	10/25 (40.0)			
		39/54 (72.2)	15/54 (27.8)				44/54 (81.5)	10/54 (18.5)			
<40 years or >60 years (*n* = 41)	41/95 (43.2)	24/63 (38.1)	17/32 (53.1)				26/70 (37.1)	15/25 (60.0)			
		24/41 (58.5)	17/41 (41.5)				26/41 (63.4)	15/41 (36.6)			
**Sex -No., %**				**0.195**	**0.142**	**0.171**			**0.360**	**0.099**	**0.338**
Female (*n* = 42)	42/95 (44.2)	31/63 (49.2)	11/32 (34.4)				33/70 (44.2)	9/25 (36.0)			
		31/42 (73.8)	11/42 (26.2)				33/42 (78.6)	9/42 (21.4)			
Male (*n* = 53)	53/95 (55.8)	32/63 (50.8)	21/32 (65.6)				3**7**/70 (55.8)	16/25 (64.0)			
		32/53 (60.4)	21/53 (39.6)				37/53 (69.8)	16/53 (30.2)			
**Highest temperature during hospitalization -No., %**				**0.002**	**0.362**	**<0.001**			**0.001**	**0.383**	**<0.001**
<37 °C (*n* = 8)	8/95 (8.4)	7/63 (11.1)	1/32 (3.1)				8/70 (11.4)	0/25 (0.0)			
		7/8 (87.5)	1/8 (12.5)				8/8 (100.0)	0/8 (0.0)			
37–38 °C (*n* = 19)	19/95 (20.0)	17/63 (27.0)	2/32 (6.3)				17/70 (24.3)	2/25 (8.0)			
		17/19 (89.5)	2/19 (10.5)				17/19 (89.5)	2/19 (10.5)			
38–39 °C (*n* = 35)	35/95 (36.8)	25/63 (39.7)	10/32 (31.3)				29/70 (41.4)	6/25 (24.0)			
		25/35 (71.4)	10/35 (28.6)				29/35 (82.9)	6/35 (17.1)			
>39 °C (*n* = 33)	33/95 (34.7)	14/63 (22.2)	19/32 (59.4)				16/70 (22.9)	17/25 (68.0)			
		14/33 (42.4)	19/33 (57.6)				16/33 (48.5)	17/33 (51.5)			

*P* values denoted the comparison between non-severe cases and severe cases

Kendall rank correlation coefficient (represented by R) was adopted to measure the degree of rank correlation between the fitness and each variable

#### Age

At first, we grouped patients into three groups (<40 years group, 40–60 years group, and >60 years group) according to their age as shown by Table 1.

Of all the 95 patients, the youngest one was 23 years old, the oldest one was 88 years old. The median age was 49.0 years (IQR, 39.0 to 58.0). 24 (25.3%) patients were aged below 40 years. 54 (56.8) patients were aged between 40 and 60 years old. 17 (17.9) patients were aged above 60 years old.

For<40 years group, 41.7% of the patients were severe cases and 33.3% of the patients reached the composite endpoint. For 40–60 years group, 27.8% of the patients were severe cases and 18.5% of the patients reached the composite endpoint. For >60 years group, 41.2% of the patients were severe cases and 41.2% of the patients reached the composite endpoint. We can see that patients aged between 40 and 60 years had lower risk of being severe cases or reaching the composite endpoint when compared with other groups. However, the *P* values were greater than 0.05. (P1 = 0.376, P2 = 0.120).

We further regrouped patients into two groups (<40 years or >60 years group, and 40–60 years group) in view of above results. As shown by Table 1, <40 years or >60 years group was related to higher risk of reaching the composite endpoint (R2 = 0.203, P4 = 0.049).

#### Gender

As shown by Table 1, 44.2% of patients were female, and 55.8% of patients were male.

For female group, 26.2% of the the patients were severe cases, and 21.4% of the patients reached the composite endpoint. For male group, 39.6% of the patients were severe cases, and 30.2% of the patients reached the endpoint. However, the *P* values were greater than 0.05. (P1 = 0.195, P2 = 0.360, P3 = 0.171, P4 = 0.338).

#### Temperature

As shown by Table 1, we grouped patients into four groups according to their highest temperature during hospitalization.

For <37 °C group, 12.5% of the patients were severe cases, and no patients reached the composite endpoint. For 37–38 °C group, 10.5% of the the patients were severe cases, and 10.5% of the patients reached the composite endpoint. For 38–39 °C group, 28.6% of the the patients were severe cases, and 17.1% of the patients reached the composite endpoint. For >39 °C, 57.6% of the the patients were severe cases, and 51.5% of the patients reached the composite endpoint. (P1 = 0.002, P2 = 0.001).

Higher temperature was related to severe 2019 novel coronavirus pneumonia and composite endpoint. (R1 = 0.362, R2 = 0.383, P3<0.001, P4<0.001).

### Laboratory findings

The Laboratory findings are shown in Table 2.

**Table 2:**
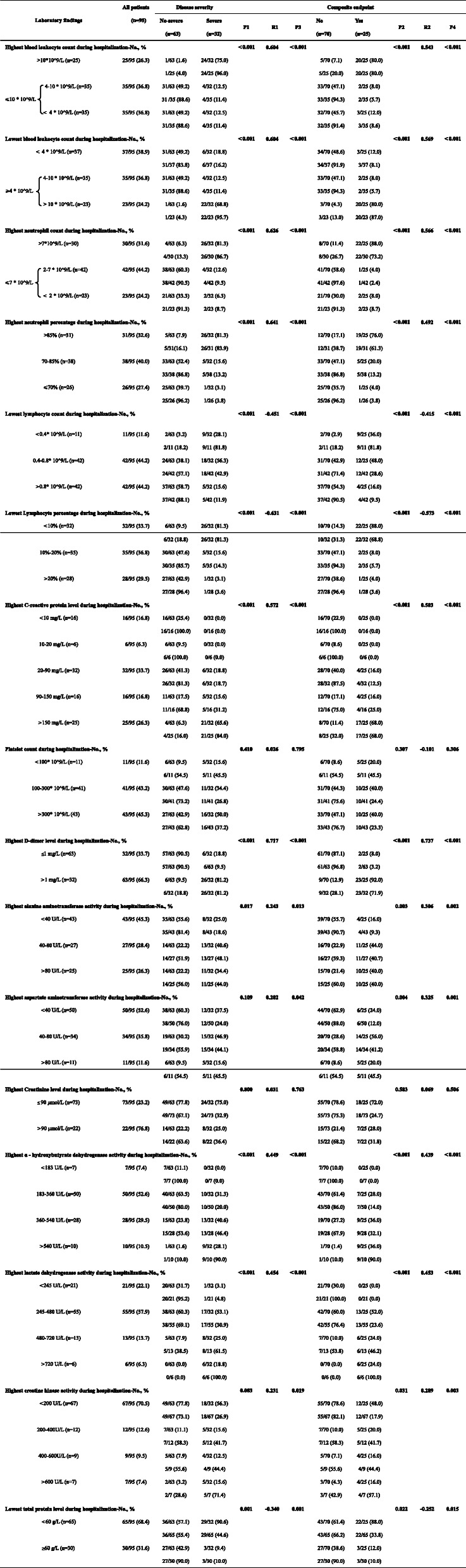
Laboratory findings of 95 patients with 2019 novel coronavirus pneumonia

#### Blood leukocyte count

##### Highest blood leukocyte count

As shown by Table 2, we first grouped patients into two groups according to their highest blood leukocyte count during hospitalization. The ≤10 * 10^9/L group were further divided into two subgroups according to their lowest blood leukocyte count during hospitalization.

For > 10*10^9/L group, 96.0% of the patients were severe cases, and 80.0% of the patients reached the composite endpoint. For 4–10 * 10^9/L group, 11.4% of the patients were severe cases, and 5.7% of the patients reached the composite endpoint. For < 4 * 10^9/L group, 11.4% of the patients were severe cases, and 8.6% of the patients reached the composite endpoint. (P1<0.001, P2<0.001).

> 10*10^9/L group were strongly related to severe 2019 novel coronavirus pneumonia and composite endpoint. (R1 = 0.604, P3<0.001, R2 = 0.543, P4<0.001).

##### Lowest blood leukocyte count

As shown by Table 2, we first grouped patients into two groups according to their lowest blood leukocyte count during hospitalization. The ≥4 * 10^9/L group were further divided into two subgroups according to their highest blood leukocyte count during hospitalization.

For<4 * 10^9/L group, 16.2% of the patients were severe cases, and 8.1% of the patients reached the composite endpoint. For 4–10 * 10^9/L group, 11.4% of the patients were severe cases, and 5.7% of the patients reached the composite endpoint. For > 10*10^9/L group, 95.7% of the patients were severe cases, and 87.0% of the patients reached the composite endpoint. (P1<0.001, P2<0.001).

> 10*10^9/L group were strongly related to severe 2019 novel coronavirus pneumonia and composite endpoint. (R1 = 0.604, P3<0.001, R2 = 0.569, P4<0.001).

#### Neutrophils

##### Highest neutrophil count

As shown by Table 2, we first grouped patients into two groups according to their highest neutrophil count during hospitalization. The ≤7 * 10^9/L group were further divided into two subgroups according to their lowest neutrophil count during hospitalization.

For > 7*10^9/L group, 86.7% of the patients were severe cases, and 73.2% of the patients reached the composite endpoint. For 2–7 * 10^9/L group, 9.5% of the patients were severe cases, and 2.4% of the patients reached the composite endpoint. For < 2 * 10^9/L group, 8.7% of the patients were severe cases, and 8.7% of the patients reached the composite endpoint. (P1<0.001, P2<0.001).

> 7*10^9/L group were strongly related to severe 2019 novel coronavirus pneumonia and composite endpoint. (R1 = 0.626, P3<0.001, R2 = 0.566, P4<0.001).

##### Highest neutrophil percentage

As shown by Table 2, we grouped patients into three groups according to their highest neutrophil percentage during hospitalization.

For > 85% group, 83.9% of the patients were severe cases, and 61.3% of the patients reached the composite endpoint. For 70–85% group, 13.2% of the patients were severe cases, and 13.2% of the patients reached the composite endpoint. For ≤70% group, 3.8% of the patients were severe cases, and 3.8% of the patients reached the composite endpoint. (P1<0.001, P2<0.001).

Higher neutrophil percentage was strongly related to severe 2019 novel coronavirus pneumonia and composite endpoint. (R1 = 0.641, P3<0.001, R2 = 0.492, P4<0.001).

#### Lymphocyte

##### Lowest lymphocyte count

As shown by Table 2, we grouped patients into three groups according to their Lowest lymphocyte count during hospitalization.

For<0.4* 10^9/L group, 81.8% of the patients were severe cases, and 81.8% of the patients reached the composite endpoint. For 0.4–0.8* 10^9/L group, 42.9% of the patients were severe cases, and 28.6% of the patients reached the composite endpoint. For >0.8* 10^9/L group, 11.9% of the patients were severe cases, and 9.5% of the patients reached the composite endpoint. (P1<0.001, P2<0.001).

Lower lymphocyte count was strongly related to severe 2019 novel coronavirus pneumonia and composite endpoint. (R1 = -0.451, P3<0.001, R2 = -0.415, P4<0.001).

##### Lowest lymphocyte percentage

As shown by Table 2, we grouped patients into three groups according to their Lowest lymphocyte percentage during hospitalization.

For<10% group, 81.3% of the patients were severe cases, and 68.8% of the patients reached the composite endpoint. For 10–20% group, 14.3% of the patients were severe cases, and 5.7% of the patients reached the composite endpoint. For>20% group, 3.6% of the patients were severe cases, and 3.6% of the patients reached the composite endpoint. (P1<0.001, P2<0.001).

Lower lymphocyte percentage was strongly related to severe 2019 novel coronavirus pneumonia and composite endpoint. (R1 = − 0.631, P3<0.001, R2 = − 0.573, P4<0.001).

#### C-reactive protein level

As shown by Table 2, we grouped patients into five groups according to their highest C-reactive protein level during hospitalization.

For<10 mg/L and 10–20 mg/L groups, no patients were severe cases, and no patients reached the composite endpoint. For 20–90 mg/L group, 18.7% of the patients were severe cases, and 12.5% of the patients reached the composite endpoint. For 90–150 mg/L group, 31.2% of the patients were severe cases, and 25.0% of the patients reached the composite endpoint. For>150 mg/L group, 84.0% of the patients were severe cases, and 68.0% of the patients reached the composite endpoint. (P1<0.001, P2<0.001).

Higher C-reactive protein level was strongly related to severe 2019 novel coronavirus pneumonia and composite endpoint. (R1 = 0.572, P3<0.001, R2 = 0.503, P4<0.001).

#### Platelet count

As shown by Table 2, we grouped patients into three groups according to their Platelet count during hospitalization.

For<100* 10^9/L groups, 45.5% of patients were severe cases, and 45.5% patients reached the composite endpoint. For 100–300* 10^9/L group, 26.8% of the patients were severe cases, and 24.4% of the patients reached the composite endpoint. For >300* 10^9/L group, 37.2% of the patients were severe cases, and 23.3% of the patients reached the composite endpoint. However, the *P* values were greater than 0.05. (P1 = 0.410, P2 = 0.307, P3 = 0.795, P4 = 0.306).

#### Highest D-dimer level

As shown by Table 2, we grouped patients into two groups according to their highest D-dimer level during hospitalization.

For ≤1 mg/L group, 9.5% of patients were severe cases, and 3.2% patients reached the composite endpoint. For>1 mg/L group, 81.2% of the patients were severe cases, and 71.9% of the patients reached the composite endpoint. (P1<0.001, P2<0.001).

Higher D-dimer level was strongly related to severe 2019 novel coronavirus pneumonia and composite endpoint. (R1 = 0.717, P3<0.001,R2 = 0.737,P4<0.001).

#### Highest alanine aminotransferase activity

As shown by Table 2, we grouped patients into three groups according to their highest alanine aminotransferase activity during hospitalization.

For<40 U/L group, 18.6% of patients were severe cases, and 9.3% patients reached the composite endpoint. For 40–80 U/L group, 48.1% of the patients were severe cases, and 40.7% of the patients reached the composite endpoint. For>80 U/L group, 44.0% of the patients were severe cases, and 40.0% of the patients reached the composite endpoint. (P1 = 0.017, P2 = 0.003).

Higher alanine aminotransferase activity was related to severe 2019 novel coronavirus pneumonia and composite endpoint. (R1 = 0.243, P3 = 0.013, R2 = 0.306, P4 = 0.002).

#### Highest aspartate aminotransferase activity

As shown by Table 2, we grouped patients into three groups according to their highest aspartate aminotransferase activity during hospitalization.

For<40 U/L group, 24% of patients were severe cases, and 12% patients reached the composite endpoint. For 40–80 U/L group, 44.1% of the patients were severe cases, and 41.2% of the patients reached the composite endpoint. For>80 U/L group, 45.5% of the patients were severe cases, and 45.5% of the patients reached the composite endpoint. (P1 = 0.109, P2 = 0.004).

Higher aspartate aminotransferase activity was related to severe 2019 novel coronavirus pneumonia and composite endpoint. (R1 = 0.202, P3 = 0.042, R2 = 0.325, P4 = 0.001).

#### Highest Creatinine level

As shown by Table 2, we grouped patients into two groups according to their highest Creatinine level during hospitalization.

For≤90 μmol/L group, 32.9% of the patients were severe cases, and 24.7% of the patients reached the composite endpoint. For>90 μmol/L group, 36.4% of the patients were severe cases, and 31.8% of the patients reached the composite endpoint. However, the *P* values were greater than 0.05. (P1 = 0.800, P2 = 0.583, P3 = 0.763, P4 = 0.506).

#### Highest α - hydroxybutyrate dehydrogenase activity

As shown by Table 2, we grouped patients into four groups according to their highest α - hydroxybutyrate dehydrogenase activity during hospitalization.

For<183 U/L group, no patients were severe cases, and no patients reached the composite endpoint. For 183–360 U/L group, 20.0% of the patients were severe cases, and 14.0% of the patients reached the composite endpoint. For 360–540 U/L group, 46.4% of the patients were severe cases, and 32.1% of the patients reached the composite endpoint. For>540 U/L group, 90.0% of patients were severe cases, and 90.0% patients reached the composite endpoint. (P1<0.001, P2<0.001).

Higher α - hydroxybutyrate dehydrogenase activity correlated strongly with severe 2019 novel coronavirus pneumonia and composite endpoint. (R1 = 0.449, P3<0.001, R2 = 0.439, P4<0.001).

#### Highest lactate dehydrogenase activity

As shown by Table 2, we grouped patients into four groups according to their highest lactate dehydrogenase activity during hospitalization.

For<245 U/L group, 4.8% of the patients were severe cases, and none of the patients reached the composite endpoint. For 245–480 U/L group, 30.9% of the patients were severe cases, and 23.6% of the patients reached the composite endpoint. For 480–720 U/L group, 61.5% of the patients were severe cases, and 46.2% of the patients reached the composite endpoint. For>720 U/L group, all the patients were severe cases, and all the patients reached the composite endpoint. (P1<0.001, P2<0.001).

Higher lactate dehydrogenase activity correlated strongly with severe 2019 novel coronavirus pneumonia and composite endpoint. (R1 = 0.454, P3<0.001, R2 = 0.453, P4<0.001).

#### Highest creatine kinase activity

As shown by Table 2, we grouped patients into four groups according to their highest creatine kinase activity during hospitalization.

For<200 U/L group, 26.9% of the patients were severe cases, and 17.9% of the patients reached the composite endpoint. For 200-400 U/L group, 41.7% of the patients were severe cases, and 41.7% of the patients reached the composite endpoint. For 400-600 U/L group, 44.4% of the patients were severe cases, and 44.4% of the patients reached the composite endpoint. For>600 U/L group, 71.4% of the patients were severe cases, and 57.1% of the patients reached the composite endpoint. (P1 = 0.083, P2 = 0.031).

Higher creatine kinase activity correlated strongly with severe 2019 novel coronavirus pneumonia and composite endpoint. (R1 = 0.231, P3 = 0.019, R2 = 0.289, P4 = 0.003).

#### Lowest total protein level

As shown by Table 2, we grouped patients into two groups according to their Lowest total protein level during hospitalization.

For<60 g/L group, 44.6% of the patients were severe cases, and 33.8% of patients reached the composite endpoint. For ≥60 g/L group, 10.0% of the patients were severe cases, and 10.0% of the patients reached the composite endpoint. (P1 = 0.001, P2 = 0.022).

Lower total protein level correlated with severe 2019 novel coronavirus pneumonia and composite endpoint. (R1 = − 0.340, P3 = 0.001, R2 = − 0.252, P4 = 0.015).

## Discussion

According to the results of our study, higher temperature, blood leukocyte count, neutrophil count, neutrophil percentage, C-reactive protein level, D-dimer level, alanine aminotransferase activity, aspartate aminotransferase activity, α - hydroxybutyrate dehydrogenase activity, lactate dehydrogenase activity and creatine kinase activity were related to severe 2019 novel coronavirus pneumonia and composite endpoint, and so were lower lymphocyte count, lymphocyte percentage and total protein level. Age below 40 or above 60 years old, male, higher Creatinine level, and lower platelet count also seemed related to severe 2019 novel coronavirus pneumonia and composite endpoint, however the *P* values were greater than 0.05, which mean under the same condition studies of larger samples are needed in the future.

Different from the results of previous studies [2, 9, 10], our study indicated that severe 2019 novel coronavirus pneumonia and composite endpoint had more to do with leukocytosis rather than leukopenia. This difference is mainly because of the different inclusion criteria and evaluation index we used. The leukocytosis was unlikely to be caused by bacterial infection because we excluded common bacteria or viruses associated with community-acquired pneumonia and procalcitonin level of all patients in our study was no greater than 0.5 ng/ml. We think that the leukocytosis was a reflection of excessive inflammation. The excessive inflammation was also reflected by the much higher C-reactive protein level of patients with severe 2019 novel coronavirus pneumonia as shown by Table 2.

In our study, the leukocytosis was always accompanied by an increase in neutrophil count and neutrophil percentage. That makes sense because the overwhelming majority of blood leukocyte was neutrophils, and the highest percentage of neutrophils was 97.5% in our study. As described above, neutrophilia was related to severe 2019 novel coronavirus pneumonia and composite endpoint. However, Up to now most of the research have focused on the role of lymphocyte in 2019 novel coronavirus pneumonia. We think that the role of neutrophils in 2019 novel novel coronavirus pneumonia shouldn’t be ignored and further related researches are needed in the future.

The leukocytosis was also accompanied by a decrease in lymphocyte count and lymphocyte percentage. It should be noted that leukocytosis was not incompatible with lymphopenia, and lymphopenia could occur both when blood leukocyte count incarease and decrease. Consistent with previous studies [11], lymphopenia was more severe and common in patients with severe 2019 novel coronavirus pneumonia. As suggested, the lymphopenia was probably caused by the translocation of lymphocyte from peripheral blood to lungs [11].

We also evaluated the Coagulation, liver and renal function of patients with 2019 novel coronavirus pneumonia.

In light of our observation, D-dimer was the most common abnormal coagulation index. As described above, higher D-dimer level was strongly related to severe 2019 novel coronavirus pneumonia and composite endpoint (R1 = 0.717, P1<0.001, R2 = 0.737, P<0.001). The increased D-dimer level reflected a hypercoagulable state, which might promote pulmonary microthrombosis. This hypothesis need to be further confirmed.

Alanine aminotransferase activity and aspartate aminotransferase activity were used to evaluate liver function in our study. As shown by Table 2, about half of the patients were found liver fuction injury, and liver fuction injury was related to severe 2019 novel coronavirus pneumonia and composite endpoint. However we didn’t see an obvious difference between 40 and 80 U/L group and >80 U/L group, and most of the liver fucntion were temporary and reversible. That means the liver function injury might be caused by multiple factors: immunity, inflammation and drugs.

Creatinine level was used to evaluate renal function in our study. A recent research indicated that the 2019 novel coronavirus was found in patients’ urine [12]. However, we didn’t see an obvious difference in creatinine level between severe group and non-severe group. Therefore, the influence of 2019 novel coronavirus on renal function need to be further studied though the 2019 novel coronavirus was found in patients’ urine.

Besides, higherα- hydroxybutyrate dehydrogenase activity, lactate dehydrogenase activity and creatine kinase activity were related to severe cases and reach of composite endpoint. In our study, 92.6% of the patients had an increase in α- hydroxybutyrate dehydrogenase activity, 77.9% of the patients had an increase in lactate dehydrogenase activity, and 29.5% of the patients had an increase in creatine kinase activity. α- hydroxybutyrate dehydrogenase activity, lactate dehydrogenase activity and creatine kinase activity were usually used to evalue the degree of myocardial injury. However, we had no direct radiological or histopathological evidence to prove that myocardial injury really happened. Thus the influence of 2019 novel coronavirus on heart also need to be further studied.

In addition we used the total protein level to evaluate the nutritional condition of patients with 2019 novel coronavirus pneumonia. According to our study, about 68.4% of the patients had a decrease in total protein level. Lower total protein level correlated with severe 2019 novel coronavirus pneumonia and composite endpoint. The decrease in total protein level reflected a high consumption state of nutrition, which suggested that patients with 2019 novel coronavirus pneumonia needed more nutrition during hospitalization.

Moreover, we compared the clinical characteristics and laboratory findings between death group and survival group as shown by Supplementary Table 1 and Supplementary Table 2. Age above 60 years old, male, blood leukocyte count > 10*10^9/L, neutrophil count > 7*10^9/L, neutrophil percentage > 85%, lymphocyte count <0.4* 10^9/L, lymphocyte percentage<10%, C-reactive protein level >150 mg/L, D-dimer level >1 mg/L, α - hydroxybutyrate dehydrogenase activity>540 U/L and lactate dehydrogenase activity>720 U/L were obviously related to a consequence of death.

In some degree, our research was different from pevious similiar studies [2, 9, 10]. Firstly we adopted a different inclusion criteria:patients with chronic or severe underlying diseases were excluded as described above. Secondly we applied different evaluation index:when assessing the relationship between candidates with the severity of 2019 novel coronavirus pneumonia, we used the highest or lowest level of candidates during hospitalization while the previous studies mainly used the fixed value of candidates on admission. We adopted different inclusion criteria and evaluation index for the following reasons: Firstly, chronic or severe underlying diseases could make it difficult to fully assess the role of 2019 novel coronavirus in 2019 novel coronavirus pneumonia without too much interference. Secondly, the laboratory findings of patients on admission could be unrepresentative because laboratory findings may vary depending on patient’s condition, and many cases that reached composite endpoint during hospitalization could be non-severe cases on admission.

However, there were also some limitaions of our study. The main limitations included the reseach type (Single center retrospective study) and a lack of cases. And it was not credible to conduct a multivariate analysis because of the lack of cases in our study.

Above are the main points of our study.

## Conclusion

Our study was different from pevious similiar studies because of the different inclusion criteria and evaluation index we applied, which offered another perspective on the relationship between various factors and 2019 novel coronavirus pneumonia.

Different from previous study we found that severe 2019 novel coronavirus pneumonia and composite endpoint had more to do with leukocytosis rather than leukopenia. Leukocytosis was always accompanied by an increase in neutrophil count and neutrophil percentage. The role of neutrophils in 2019 novel coronavirus pneumonia shouldn’t be ignored and further related researches are needed in the future. Consistent with previous studies, lymphopenia was found more severe and common in patients with severe 2019 novel coronavirus pneumonia. The lymphopenia was probably caused by the translocation of lymphocyte from peripheral blood to lungs [11].

Besides higher D-dimer level and impaired liver function were related to severe 2019 novel coronavirus pneumonia and composite endpoint. The increased D-dimer level reflected a hypercoagulable state, which might promote pulmonary microthrombosis. This hypothesis need to be further confirmed. Most of the liver fucntion were temporary and reversible, and the liver function injury might be caused by multiple factors: immunity, inflammation and drugs. However, we didn’t see an obvious difference in creatinine level between severe group and non-severe group.

In addition, higherα- hydroxybutyrate dehydrogenase activity, lactate dehydrogenase activity and creatine kinase activity were related to severe cases and reach of composite endpoint. The influence of 2019 novel coronavirus on heart also need to be further studied.

Furthermore the decrease in total protein level reflected a high consumption state of nutrition, which suggested that patients with 2019 novel coronavirus pneumonia need more nutrition during hospitalization.

In summary, multiple factors were found related to the severity of 2019 novel coronavirus pneumonia and composite endpoint. More related studies are needed in the future.

## Supplementary information

**Additional file 1 : Supplementary Table 1.** Clinical characteristics of 95 patients with 2019 novel coronavirus pneumonia. **Supplementary Table 2.** Laboratory findings of 95 patients with 2019 novel coronavirus pneumonia.

## Data Availability

All data generated or analysed during this study are included in this published article [and its supplementary information files].

## Abbreviations

SARS-CoV: Severe acute respiratory syndrome coronavirus
MERS-COV: Middle East respiratory syndrome coronavirus
ICU: Intensive care unit
IQR: Interquartile ranges
SPSS: Statistical product and service solutions
