# Supplementary material for: Analysis of clinical characteristics and laboratory findings of 95 cases of 2019 novel coronavirus pneumonia in Wuhan, China: a retrospective analysis
Source: Respir Res. 2020 Mar 26;21:74. doi: 10.1186/s12931-020-01338-8 (PMC7099829; doi:10.1186/s12931-020-01338-8)
Supplement: Supplementary file 1 — Additional file 1 : Supplementary Table 1. Clinical characteristics of 95 patients with 2019 novel coronavirus pneumonia. Supplementary Table 2. Laboratory findings of 95 patients with 2019 novel coronavirus pneumonia. [file 12931_2020_1338_MOESM1_ESM.docx]

**Supplementary table 1. Clinical characteristics of 95 patients with 2019 novel coronavirus pneumonia**

| **Clinical characteristics** | **All patients**  **(n=95)** | **Outcome** | |  | | |
| --- | --- | --- | --- | --- | --- | --- |
|  |  | **Survival**  **(n=89)** | **Death**  **(n=6)** | **P1** | **R** | **P2** |
| **Age, Median (range)-years** | 49.0 (39.0-58.0) | 49.0 (39.5-57.0) | 66.0 (38.3-76.8) | **0.085** | **0.148** | **0.084** |
| **Age groups-No., %** |  |  |  | **0.002** | **0.144** | **0.145** |
| ＜40years (n=24) | 24/95 (25.3) | 22/89 (24.7) | 2/6 (33.3) |  |  |  |
|  |  | 22/24 (91.7) | 2/24 (8.3) |  |  |  |
| 40-60 years (n=54) | 54/95 (56.8) | 54/89 (60.7) | 0/6 (0.0) |  |  |  |
|  |  | 54/54 (100.0) | 0/54 (0.0) |  |  |  |
| ＞60 years (n=17) | 17/95 (17.9) | 13/89 (14.6) | 4/6 (66.7) |  |  |  |
|  |  | 13/17 (76.5) | 4/17 (23.5) |  |  |  |
| **Age groups-No., %** |  |  |  | **0.005** | **0.298** | **0.004** |
| 40-60 years (n=54) | 54/95 (56.8) | 54/89 (60.7) | 0/6 (0.0) |  |  |  |
|  |  | 54/54 (100.0) | 0/54 (0.0) |  |  |  |
| ＜40 years or ＞60 years (n=41) | 41/95 (43.2) | 35/89 (39.3) | 6/6 (100.0) |  |  |  |
|  |  | 35/41 (85.4) | 6/41 (14.6) |  |  |  |
| **Sex -No., %** |  |  |  | **0.032** | **0.231** | **0.025** |
| Female (n=42) | 42/95 (44.2) | 42/89 (47.2) | 0/6 (0.0) |  |  |  |
|  |  | 42/42 (100.0) | 0/42 (0.0) |  |  |  |
| Male (n=53) | 53/95 (55.8) | 47/63 (52.8) | 6/6 (100.0) |  |  |  |
|  |  | 47/53 (88.7) | 6/53 (11.3) |  |  |  |
| **Highest temperature during hospitalization -No., %** | |  |  | **0.293** | **0.182** | **0.057** |
| ＜37 ℃ (n=8) | 8/95 (8.4) | 8/89 (9.0) | 0/6 (0.0) |  |  |  |
|  |  | 8/8 (100.0) | 0/8 (0.0) |  |  |  |
| 37-38 ℃ (n=19) | 19/95 (20.0) | 19/89 (21.3) | 0/6 (0.0) |  |  |  |
|  |  | 19/19 (100.0) | 0/19 (0.0) |  |  |  |
| 38-39 ℃ (n=35) | 35/95 (36.8) | 33/89 (37.1) | 2/6 (33.3) |  |  |  |
|  |  | 33/35 (94.3) | 2/35 (5.7) |  |  |  |
| ＞39 ℃ (n=33) | 33/95 (34.7) | 29/89 (32.6) | 4/6 (66.7) |  |  |  |
|  |  | 29/33 (87.9) | 4/33 (12.1) |  |  |  |

P values denoted the comparison between non-severe cases and severe cases.

Kendall rank correlation coefficient (represented by R) was adopted to measure the degree of rank correlation between the fitness and each variable.

**Supplementary table 2. Laboratory findings of 95 patients with 2019 novel coronavirus pneumonia**

| **Laboratory findings** | **All patients**  **(n=95)** | **Outcome** | |  | | |
| --- | --- | --- | --- | --- | --- | --- |
|  |  | **Survival**  **(n=89)** | **Death**  **(n=6)** | **P1** | **R** | **P2** |
| **Highest blood leukocyte count during hospitalization-No., %** | |  |  | **＜0.001** | **0.333** | **0.001** |
| >10*10^9/L (n=25) | 25/95 (26.3) | 19/63 (21.3) | 6/6 (100.0) |  |  |  |
|  |  | 19/25 (76.0) | 6/25 (24.0) |  |  |  |
| 4-10 * 10^9/L (n=35) | 35/95 (36.8) | 35/63 (39.3) | 0/32 (0.0) |  |  |  |
| ≤10 * 10^9/L |  | 35/35 (100.0) | 0/35 (0.0) |  |  |  |
| ＜ 4 * 10^9/L (n=35) | 35/95 (36.8) | 35/63 (39.3) | 0/32 (0.0) |  |  |  |
|  |  | 35/35 (100.0) | 0/35 (0.0) |  |  |  |
| **Lowest blood leukocyte count during hospitalization-No., %** | |  |  | **＜0.001** | **0.344** | **＜0.001** |
| ＜ 4 * 10^9/L (n=37) | 37/95 (38.9) | 37/89 (41.6) | 0/6 (18.8) |  |  |  |
|  |  | 37/37 (100.0) | 0/37 (0.0) |  |  |  |
| 4-10 * 10^9/L (n=35) | 35/95 (36.8) | 35/89 (39.3) | 0/6 (12.5) |  |  |  |
| ≥4 * 10^9/L |  | 35/35 (100.0) | 0/35 (0.0) |  |  |  |
| > 10 * 10^9/L (n=23) | 23/95 (24.2) | 17/89 (19.1) | 6/6 (68.8) |  |  |  |
|  | | 17/23 (73.9) | 6/23 (26.1) |  |  |  |
| **Highest neutrophil count during hospitalization-No., %** | |  |  | **0.001** | **0.313** | **0.001** |
| >7*10^9/L (n=30) | 30/95 (31.6) | 24/89 (27.0) | 6/6 (100.0) |  |  |  |
|  |  | 24/30 (80.0) | 6/30 (20.0) |  |  |  |
| 2-7 * 10^9/L (n=42) | 42/95 (44.2) | 42/89 (47.2) | 0/6 (0.0) |  |  |  |
| ≤7 * 10^9/L |  | 38/42 (100.0) | 0/42 (0.0) |  |  |  |
| ＜ 2 * 10^9/L (n=23) | 23/95 (24.2) | 23/89 (25.8) | 0/6 (0.0) |  |  |  |
|  |  | 23/23 (100.0) | 0/23 (0.0) |  |  |  |
| **Highest neutrophil percentage during hospitalization-No., %** | |  |  | **0.001** | **0.305** | **0.002** |
| >85% (n=31) | 31/95 (32.6) | 25/89 (28.1) | 6/6 (100.0) |  |  |  |
|  |  | 25/31(80.6) | 6/31 (19.4) |  |  |  |
| 70-85% (n=38) | 38/95 (40.0) | 38/89 (42.7) | 0/6 (0.0) |  |  |  |
|  |  | 38/38 (100.0) | 0/38 (0.0) |  |  |  |
| ≤70% (n=26) | 26/95 (27.4) | 26/89 (29.2) | 0/6 (0.0) |  |  |  |
|  |  | 26/26 (100.0) | 0/26 (0.0) |  |  |  |
| **Lowest lymphocyte count during hospitalization-No., %** | |  |  | **0.009** | **-0.218** | **0.028** |
| ＜0.4* 10^9/L (n=11) | 11/95 (11.6) | 8/89 (9.0) | 3/6 (50.0) |  |  |  |
|  |  | 8/11 (72.7) | 3/11 (27.3) |  |  |  |
| 0.4-0.8* 10^9/L (n=42) | 42/95 (44.2) | 40/89 (44.9) | 2/6 (33.3) |  |  |  |
|  |  | 40/42 (95.2) | 2/42 (4.8) |  |  |  |
| ＞0.8* 10^9/L (n=42) | 42/95 (44.2) | 41/89 (46.1) | 1/6 (16.7) |  |  |  |
|  |  | 41/42 (97.6) | 1/42 (2.4) |  |  |  |
| **Lowest Lymphocyte percentage during hospitalization-No., %** | |  |  | **0.002** | **-0.299** | **0.002** |
| ＜10% (n=32) | 32/95 (33.7) | 26/89 (29.2) | 6/6 (100.0) |  |  |  |
|  |  | 26/32 (81.3) | 6/32 (18.7) |  |  |  |
| 10%-20% (n=35) | 35/95 (36.8) | 35/89 (39.3) | 0/6 (0.0) |  |  |  |
|  |  | 35/35 (100.0) | 0/35 (0.0) |  |  |  |
| ＞20% (n=28) | 28/95 (29.5) | 28/89 (31.5) | 0/6 (0.0) |  |  |  |
|  |  | 28/28 (100.0) | 0/28 (0.0) |  |  |  |
| **Highest C-reactive protein level during hospitalization-No., %** | |  |  | **0.001** | **0.311** | **0.001** |
| ＜10 mg/L (n=16) | 16/95 (16.8) | 16/89 (18.0) | 0/6 (0.0) |  |  |  |
|  |  | 16/16 (100.0) | 0/16 (0.0) |  |  |  |
| 10-20 mg/L (n=6) | 6/95 (6.3) | 6/89 (6.7) | 0/6 (0.0) |  |  |  |
|  |  | 6/6 (100.0) | 0/6 (0.0) |  |  |  |
| 20-90 mg/L (n=32) | 32/95 (33.7) | 32/89 (36.0) | 0/6 (0.0) |  |  |  |
|  |  | 32/32 (100.0) | 0/32 (0.0) |  |  |  |
| 90-150 mg/L (n=16) | 16/95 (16.8) | 16/89 (18.0) | 0/6 (0.0) |  |  |  |
|  |  | 16/16 (100.0) | 0/16 (0.0) |  |  |  |
| ＞150 mg/L (n=25) | 25/95 (26.3) | 19/89 (21.3) | 6/6 (100.0) |  |  |  |
|  |  | 19/25 (76.0) | 6/25 (24.0) |  |  |  |
| **Platelet count during hospitalization-No., %** | |  |  | **0.346** | **-0.134** | **0.178** |
| ＜100* 10^9/L (n=11) | 11/95 (11.6) | 10/89 (11.2) | 1/6 (16.7) |  |  |  |
|  |  | 10/11 (90.9) | 1/11 (9.1) |  |  |  |
| 100-300* 10^9/L (n=41) | 41/95 (43.2) | 37/89 (41.6) | 4/6 (66.7) |  |  |  |
|  |  | 37/41 (90.2) | 4/41 (9.8) |  |  |  |
| ＞300* 10^9/L (43) | 43/95 (45.3) | 42/89 (47.2) | 1/6 (16.7) |  |  |  |
|  |  | 42/43 (97.7) | 1/43 (2.3) |  |  |  |
| **Highest D-dimer level during hospitalization-No., %** | |  |  | **0.001** | **0.364** | **＜0.001** |
| ≤1 mg/L (n=63) | 32/95 (33.7) | 63/89 (70.8) | 0/6 (0.0) |  |  |  |
|  |  | 63/63 (100.0) | 0/63 (0.0) |  |  |  |
| ＞1 mg/L (n=32) | 63/95 (66.3) | 26/89 (29.2) | 6/6 (100.0) |  |  |  |
|  |  | 26/32 (81.3) | 6/32 (18.7) |  |  |  |
| **Highest alanine aminotransferase activity during hospitalization-No., %** | | |  | **0.478** | **0.016** | **0.869** |
| ＜40 U/L (n=43) | 43/95 (45.3) | 41/89 (55.6) | 2/6 (33.3) |  |  |  |
|  |  | 41/43 (81.4) | 2/43 (18.6) |  |  |  |
| 40-80 U/L (n=27) | 27/95 (28.4) | 24/89 (22.2) | 3/6 (50.0) |  |  |  |
|  |  | 24/27 (51.9) | 3/27 (48.1) |  |  |  |
| ＞80 U/L (n=25) | 25/95 (26.3) | 24/89 (22.2) | 1/6 (16.7) |  |  |  |
|  |  | 24/25 (56.0) | 1/25 (44.0) |  |  |  |
| **Highest aspartate aminotransferase activity during hospitalization-No., %** | | |  | **0.023** | **0.236** | **0.018** |
| ＜40 U/L (n=50) | 50/95 (52.6) | 50/89 (56.2) | 0/6 (0.0) |  |  |  |
|  |  | 50/50 (100.0) | 0/50 (0.0) |  |  |  |
| 40-80 U/L (n=34) | 34/95 (35.8) | 29/89 (32.6) | 5/6 (83.3) |  |  |  |
|  |  | 29/34 (85.3) | 5/34 (14.7) |  |  |  |
| ＞80 U/L (n=11) | 11/95 (11.6) | 10/89 (11.2) | 1/6 (16.7) |  |  |  |
|  |  | 10/11 (90.9) | 1/11 (9.1) |  |  |  |
| **Highest Creatinine level during hospitalization-No., %** | |  |  | **0.135** | **0.165** | **0.109** |
| ≤ 90 μmol/L (n=73) | 73/95 (23.2) | 70/89 (78.7) | 3/6 (50.0) |  |  |  |
|  |  | 70/73 (95.9) | 3/73 (4.1) |  |  |  |
| ＞90 μmol/L (n=22) | 22/95 (76.8) | 19/89 (21.3) | 3/6 (50.0) |  |  |  |
|  |  | 19/22 (86.4) | 3/22 (13.6) |  |  |  |
| **Highest α - hydroxybutyrate dehydrogenase activity during hospitalization-No., %** | | | | **＜0.001** | **0.417** | **＜0.001** |
| ＜183 U/L (n=7) | 7/95 (7.4) | 7/89 (7.9) | 0/6 (0.0) |  |  |  |
|  |  | 7/7 (100.0) | 0/7 (0.0) |  |  |  |
| 183-360 U/L (n=50) | 50/95 (52.6) | 50/89 (56.2) | 0/6 (0.0) |  |  |  |
|  |  | 50/50 (100.0) | 0/50 (0.0) |  |  |  |
| 360-540 U/L (n=28) | 28/95 (29.5) | 28/89 (100.0) | 0/6 (0.0) |  |  |  |
|  |  | 28/28 (100.0) | 0/28 (0.0) |  |  |  |
| ＞540 U/L (n=10) | 10/95 (10.5) | 4/89 (4.5) | 6/6 (100.0) |  |  |  |
|  |  | 4/10 (40.0) | 6/10 (60.0) |  |  |  |
| **Highest lactate dehydrogenase activity during hospitalization-No., %** | | |  | **＜0.001** | **0.431** | **＜0.001** |
| ＜245 U/L (n=21) | 21/95 (22.1) | 21/89 (23.6) | 0/6 (0.0) |  |  |  |
|  |  | 21/21 (100.0) | 0/21 (0.0) |  |  |  |
| 245-480 U/L (n=55) | 55/95 (57.9) | 55/89 (61.8) | 0/6 (0.0) |  |  |  |
|  |  | 55/55 (100.0) | 0/55 (0.0) |  |  |  |
| 480-720 U/L (n=13) | 13/95 (13.7) | 12/89 (13.5) | 1/6 (16.7) |  |  |  |
|  |  | 12/13 (92.3) | 1/13 (7.7) |  |  |  |
| ＞720 U/L (n=6) | 6/95 (6.3) | 1/89 (1.1) | 5/6 (83.3) |  |  |  |
|  |  | 1/6 (16.7) | 5/6 (83.3) |  |  |  |
| **Highest creatine kinase activity during hospitalization-No., %** | |  |  | **0.115** | **0.213** | **0.031** |
| ＜200 U/L (n=67) | 67/95 (70.5) | 65/89 (73.0) | 2/6 (33.3) |  |  |  |
|  |  | 65/67 (97.0) | 2/67 (3.0) |  |  |  |
| 200-400U/L (n=12) | 12/95 (12.6) | 11/89 (12.4) | 1/6 (16.7) |  |  |  |
|  |  | 11/12 (91.7) | 1/12 (8.3) |  |  |  |
| 400-600U/L (n=9) | 9/95 (9.5) | 7/89 (7.9) | 2/6 (33.3) |  |  |  |
|  |  | 7/9 (77.8) | 2/9 (22.2) |  |  |  |
| ＞600 U/L (n=7) | 7/95 (7.4) | 6/89 (6.7) | 1/6 (16.7) |  |  |  |
|  |  | 6/7 (85.7) | 1/7 (14.3) |  |  |  |
| **Lowest total protein level during hospitalization-No., %** | |  |  | **0.661** | **-0.083** | **0.419** |
| ＜60 g/L (n=65) | 65/95 (68.4) | 60/89 (67.4) | 5/6 (83.3) |  |  |  |
|  |  | 60/65 (92.3) | 5/65 (7.7) |  |  |  |
| ≥60 g/L (n=30) | 30/95 (31.6) | 29/89 (32.6) | 1/6 (16.7) |  |  |  |
|  |  | 29/30 (96.7) | 1/30 (3.3) |  |  |  |
